# A Retrospective Study on Scropionism in Iran (2002–2011)

**Published:** 2017-05-27

**Authors:** Rouhullah Dehghani, Javad Rafinejad, Behrooz Fathi, Morteza Panjeh Shahi, Mehrdad Jazayeri, Afsaneh Hashemi

**Affiliations:** 1 Social Determinants of Health (SDH) Research Center and Department of Environmental Health, Kashan University of Medical Sciences, Kashan, Iran; 2 Department of Medical Entomology, School of Public Health, Tehran University of Medical Sciences, Tehran, Iran; 3 Department of Pharmacology, School of Veterinary Medicine, Ferdowsi University of Mashhad, Mashhad, Iran; 4 Health Center, Kashan University of Medical Sciences and Health Services, Kashan, Iran; 5 Veterinarian Medicine, Head Office of Veterinary Khorassan Razavi Province, Mashhad, Iran; 6 Center for Solid Waste Research (CSWR), Institute for Environmental Research (IER), Tehran University of Medical Sciences, Tehran, Iran

**Keywords:** Scorpion stings, Epidemiology, Iran

## Abstract

**Background:**

Scorpion sting is a common medical emergency in Iran. The epidemiological features and control of such cases vary from south to north. This review will provide new information about the epidemiology of scorpion stings in different provinces of Iran.

**Methods:**

In this descriptive retrospective study, data on scorpionism including incidence rates, mortality as well as locality from 2002 to 2011were collected.

**Results:**

Overall, 433203 victims of scorpion stings had been referred to health centers from of all of the 31 provinces. The incidence of scorpion stings in 100000 populations was from 54.8 to 66. The highest rate of scorpion stings occurred among the 25–34 yr old group. The highest incidence of scorpion stings during 2011 was observed in Khuzestan Province and the lowest number in Mazandaran Province. The peak number of human cases (scorpion stings) was recorded during May to August.

**Conclusion:**

Scorpion stings in Iran are high. It is necessary that physicians and health care professionals should be familiar with local scorpions, especially those that are potentially more dangerous than others, the effective treatment protocols, and supportive care as well as their control of scorpionism

## Introduction

Venomous animal bites and stings are one of the important health concerns in rural regions in many parts of the world (Warrell 1992). Scorpions form a large group of poisonous creatures found throughout the warm and temperate regions of the world. Nearly all medically significant species of scorpions are located in Buthidae family. Those considered most dangerous are native to Africa, Asia and the Middle East (Dehghani and Arani 2015).

The outcomes of scorpion sting may cause only localized or general pain but may also cause more severe manifestations such as shock, kidney damage and cardiac muscle injury (White 2000). Scorpions are an evolutionarily old group of animals and some 2000 species have been described from around the world (Dupre 2012). In Iran, the scorpion fauna has had a long-standing interest from scientist’s worldwide long ago paying attention of the scientists worldwide in terms of systematic, biology and ecology. Dispensation information of species is reviewed based on scientific literature until 2012. Scorpion stings were observed in all over Iran, and there are 51 species of scorpions found in different parts of the country, but only ten species are important (Dehghani et al. 2012).

Like with most creatures that use venom only for defense, scorpions seldom sting their prey, as a substitute using their pedipalps and crushing chelicera to acquire food (Vetter and Visscher 1998). Within scorpion fauna of Iran, the Buthidae family is the more frequent, with 82% of all the genera and 88.5% of all the species. Among these, the *Androctonus* genera have the main number of known medically significant species. The number species of two others families is alike. The Hemiscorpiidae family is, with 2 genus (9%) and 3 species (5.75%). In this family, the medically chief genus is *Hemiscorpius* with the main number of notorious species. Lastly, Scorpionidae is another family with 2 genus (9%) and 3 species (5.75%). The south and southwest of Iran with about 95% species of scorpions are the most heavily occupied areas in the country (Navidpour et al. 2008a,b).

Khuzestan Province is highlighted for its scorpions and scorpion sting amongst the provinces of Iran (Vazirainzadeh et al. 2012). Khuzestan with 19 species of scorpions is one of the most significant areas in terms of scorpion sting problem in the south west of Iran. In Iran, medical importance, epidemiology and geographic distribution of scorpions have been reported (Dehghani 1998, Navidpour et al. 2008a, Dehghani et al. 2012). Due to the significance of scorpion stings and the shortage of epidemiological data about this public health difficulty, the study was carried out to collect new data concerning scorpion stings in Iran. This would allow the system to arrangement strategies to decrease and scorpion stings among the inhabitants of all provinces in Iran.

The purpose of this project was to conduct a retrospective study to describe the incidence and geographic location of scorpion stings in Iran provinces, and to assess the magnitude and distribution of the problem in order to optimize prevention and treatment.

## Materials and Methods

This descriptive and retrospective study was carried out in 2012. Data was gathered based on information of Ministry of Health and Medical Education from the files of outpatient or hospitalized persons referred to the health centers and hospitals of all provinces for the last decade. The data of scorpion stings was assessed from the epidemiological aspects including: gender and ages of scorpion sting victims, antivenin therapy, the time of scorpion sting, subjected parts of body, and environmental circumstances such as rural or urban habitats. The recorded data were evaluated prospectively and statistically analyzed using Excel’s simple statistical functions, and then interpreted and presented in the form of tables and graphs

### Geographical information on Iran

Iran is the eighteenth biggest country of the world. Its borders are limited to the north, Caspian Sea, in the northwest, Armenia and Azerbaijan, the east, Afghanistan and Pakistan, in the west, Iraq and Turkey, in the northeast, Turkmenistan, and ultimate the waters of the Persian Gulf and the Sea of Oman in the south. Iran’s district is 1,648,000 Square km.

Iran has three separate geographies: (a) the foremost mountain ranges comprise the Zagros Mountains in the west and south, and the Elburz Mountains in the north. Most of these mountains are upper than 2,440 meters. Some peaks are higher than 4,268 meters in the Zagros and 5,486 meters in the Elburz, including Qolleh-ye Damavand at 5,671 meters. (b) Most of the rest of the country consists of a flat terrain that contains several closed basins and two salt deserts, the Dasht-e Kavir and the Dasht-e Lut. A lot of the flat terrain has internal drainage and is distinguished by many irregular streams, discontinuous salt lakes, and wet salt flats. (c) Lesser flat plains are situated the length of the Caspian Sea, Persian Gulf, and Sea of Oman (http://en.wikipedia.org/wiki/Geography_of_Iran).

Iran has various climates with very warm summer and cold, with some snow, winter. Winter season is usually the rainy for the entire country. The northwest is generally the coldest and among the rainiest parts of the country. The winters in Kurdistan Province and West and East Azarbaijan and Ardabil Provinces and can be harsh: temperatures from time to time drop as low as −20 °C. Snow often remains until near the beginning spring, or even later in the mountains. However the littoral regions have moderately a different weather. The Caspian Sea shoreline is moist all year round and provides a difference with the aridness of highland. The temperature can vary typically. In the summer, temperatures differ from 50 °C in the south to 1 °C in the northwest. Mean winter and summer are 5.9 °C and 37.8 °C respectively. Rainfall also varies actually, range from less than 50 mm in the southeast to about 2000mm in the Caspian region. The annually mean is about 250mm (reference http://iranto.ca/En/index.php/about-iran/geography-and-climate).

## Results

**Fig. 1.**
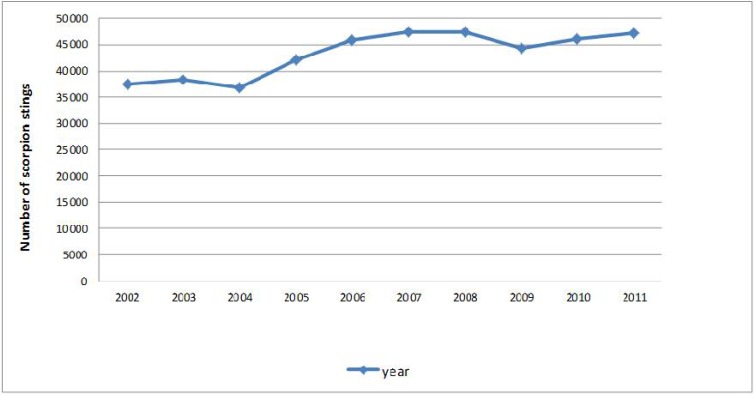
Number of scorpion stings in Iran during 2002–2011

**Fig. 2.**
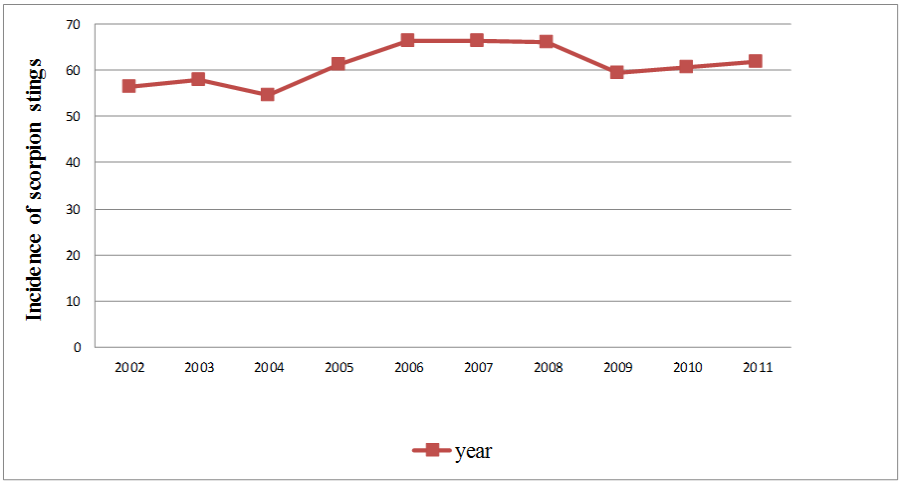
Incidence of scorpion sting in Iran during 2002–2011

**Fig. 3.**
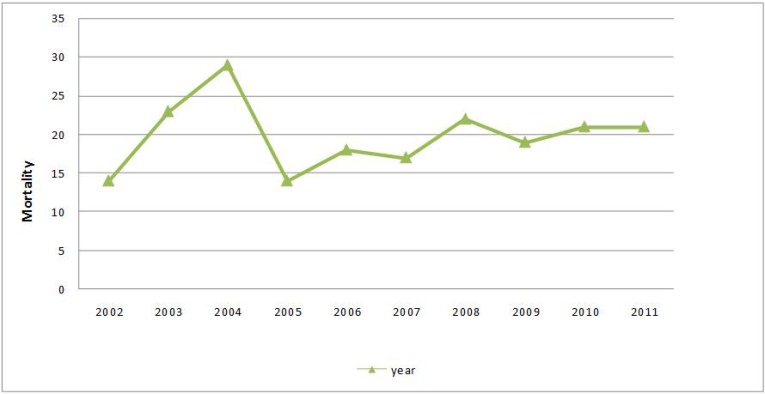
Number of human cases of scorpion stings resulted to death in Iran during 2002–2011

**Fig. 4.**
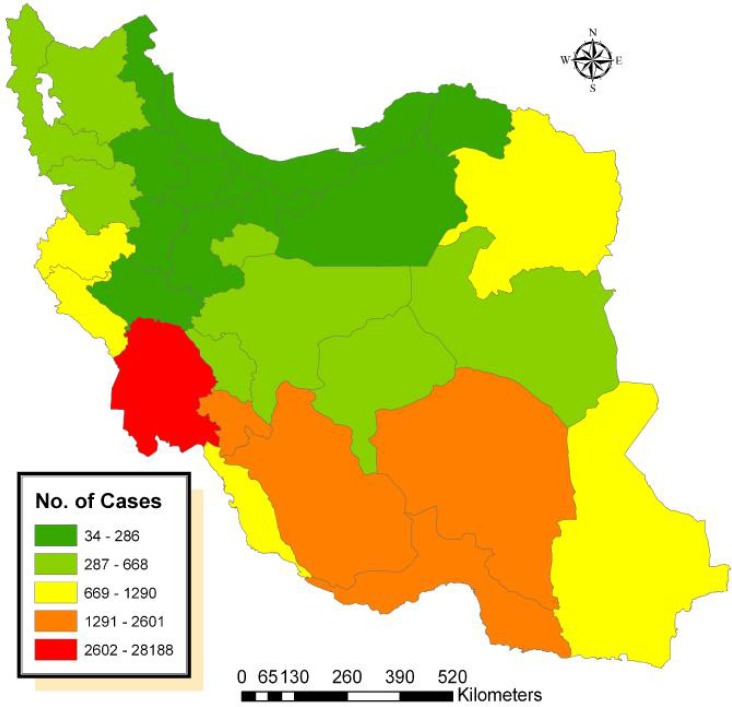
Spatial distribution map of scorpion sting cases in different Provinces of Iran, 2002–2011

**Fig. 5.**
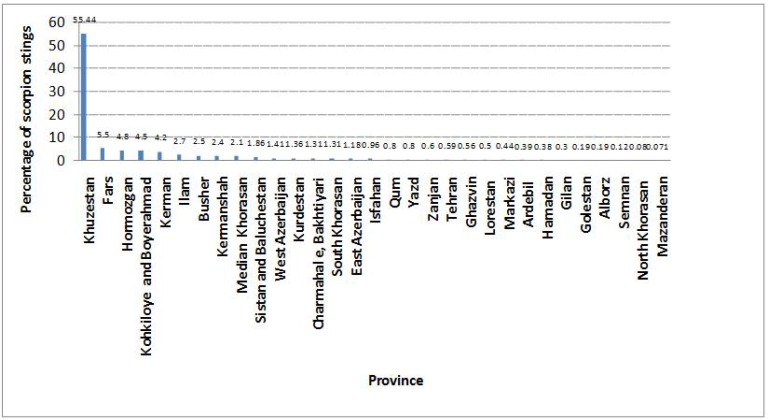
Percentage of scorpion stings according to provinces in Iran during 2002–2011

**Fig. 6.**
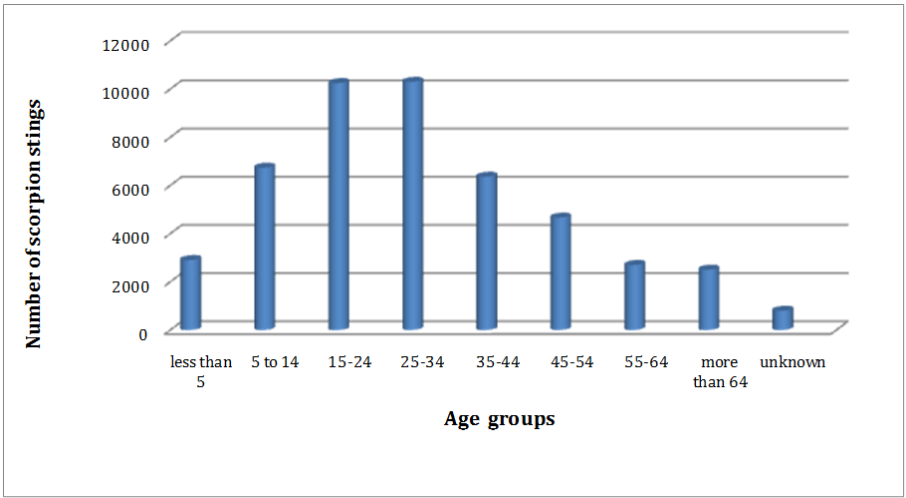
Frequency of scorpion stings according to age groups in Iran during 2002–2011

**Fig. 7.**
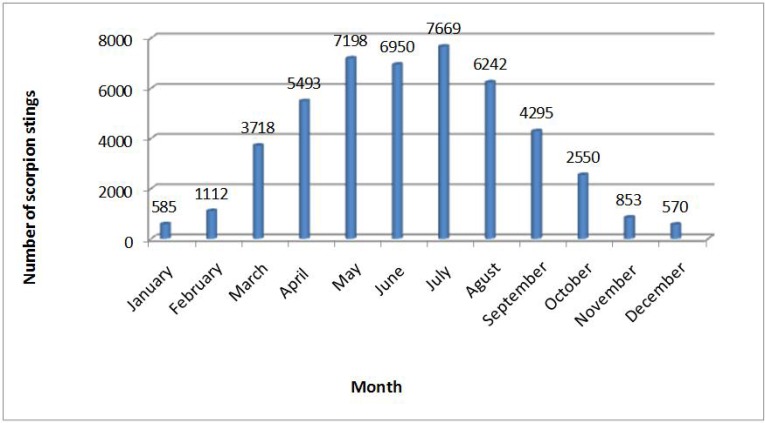
Number of scorpion stings, based on month in Iran during 2002–2011

During 2002–2011, a total of 433201 scorpion-stung patients referred to the health center and hospitals of all provinces (Fig. 1). The incidence of scorpion stings in 100000 of population was from 54.8 to 66 during 2002–2011 (Fig. 2). The highest incidence of scorpion sting cases were reported in 2008 (47510) and the lowest in 2004 (36806) in all provinces of Iran. Out of 433201 scorpion sting cases, 433003 cases (99.95%) recovered, however deaths (0.05%) were recorded during the study period (Fig. 3). The highest mortality rate of scorpion stings were occured during 2004 (29), however the lowest mortality rate of scorpion stings were reported in 2002 (14) Among all provinces of Iran highest prevalence of scorpion sting and its resulting death has been recorded from Khuzestan and the lowest in Mazandaran Provinces, respectively (Figs. 4 and 5). Only about 55.5% of scorpion stings and 38.1% the mortalities occurred in Khuzestan Province. The highest of scorpion stings after Khuzestan were reported in Fars, Hormozgan, Kohgiloye and Boyerahmad, Kerman and the rest of provinces of Iran, respectively. Based on recorded data, the rate of incidences in different provinces has increased from north to south. The incidence of scorpion sting in Iran has been calculated as 62.02 persons in 100000 during one year (March 22^nd^ 2011–March 21^st^ 2011). In general, most victims of scorpion stung people were in rural (24683: 52%), and 46 % (21803) from urban and the rest (729:2%) were unknown areas of Iran. Wholly, the number and percent of stung women and men of scorpion-stung patients were 24339 (52%) and 22896 (48%), respectively. The highest rate of scorpion stings victims are related to the 15–34 yr old (43.5%) followed by 5–14 (14.3%) and 35–44 (13.5%). The lowest rate of scorpion stings were reported among the population more than 65 yr old (5.3%) (Fig. 6). Scorpion sting were seen in all months, the highest incidence of scorpion sting cases were in July (16.3%) and the lowest in December (1.2%) in Iran (Fig. 7). Legs and hands were exposed by scorpion more than the other parts (80%), followed by head and trunk with 18%, the rest (2%) were unknown. Out of 47,235 scorpion sting cases, 47,214 cases (99.95%) recovered, however deaths (0.05%) were recorded during the 2011. Totally, 16% of victims recovered using convenience treatments without scorpion antivenin serum. However, the rest were treated by scorpion antivenin serum including intra-muscular (32849:86%) and intra-venin (5540:14%) injections and convenience treatments.

The interval hours after stings and injections were recorded as: survival was 60.9 after 0–6 h, 11.8% after 6–12 h and 6.3% after more than 12 h. Out of 21 deaths, 11 cases were not received any antivenin, and the rest were received antivenin, 10 cases of deaths occurred during 6 to 12 h and more than 12 h, after scorpion antivenin serum was injected.

## Discussion

The present study showed that the incidence of scorpion stings in 100000 of population was from 54.8 to 66 during 2002–2011. These variations were likely due to the difference of climatologic factors and preventive measures. The mean rate of sting incidences in Iran was 61.2 per 100,000 populations during the study period. In the world incidence annually, 1.2 million people are estimated with around 3250 deaths per year. Base on Chippaux and Goyffon 2010, the mean rate of sting incidences in the world in per year per 100,000 population is about 17.14 (Chippaux and Goyffon 2010). It shows scorpion sting incidence in Iran which is higher than the global average.

The important matter is that the scorpions stung every year with a resembling rate 54.8 to 66 during whole period of 2002–2011 (Table 1). The highest of scorpion stings were reported in Khuzestan and the lowest in Mazadaran. This agrees with the results of Dehghani and Fathi (2012), Labaf Ghasemi (1999) and Rafizadeh et al. (2013) (Labaf Ghasemi 1999, Dehghani et al. 2012, Rafizadeh et al. 2013). There was a difference between frequency of males (48%) and females (52%) among the patients referred to the health centers and hospitals, with scorpion stings. It means that the females were at higher risk of scorpion stings than males in all provinces of Iran. This amount is agreed with the results of Vazirianzadeh et al. 2012, Shahbazzadeh et al. 2009). Our results are not in agreed with the results in Saudi Arabia (Al-Sadoon 2003, Jarrar 2008) who reported that scorpion stings were higher in males than in females.

The highest rate of scorpion stings occurred among the 15–34 yr old people, in accordance with the findings in Kashan, central of Iran (Deghani et al. 2010), Ahvaz, south west of Iran (Emam at al. 2008). The highest incidence of scorpion sting cases during 2011 occurred in summer (44.16%). This is in agree with the studies in Iran (Rafeeazadeh et al. 2009, Deghani et al. 2010, Dehghani and Fathi 2012a), Saudi Arabia (Shahbazzadeh et al. 2009, Jarrar and Al-Rowaily 2008) and in Turkey (Emam et al. 2008, Ozkan and KAT 2005). They have reported that 49.7–93.4% of scorpion sting cases occurred in summer.

In general, a total of 84% scorpion stung persons received antivenin. This antivenin is prepared in Razi Vaccine and Serum Research Institute, Iran in a 5ml hexavalent vial of 6 species including *Hemiscorpius lepturus*, *Androctonus crassicauda*, *Mesobuthus eupeus*, *Odontobuthus doriae*, *Hottentotta saulcyi* and *Hottentotta schach* (Sanaei-Zadeh 2014). The most important health-threatening scorpions in Iran is *Androctonus crassicauda* from Buthidae family and *Hemiscorpius lepturus*, which belongs to the Hemiscorpiidae family. They are described as potentially dangerous to humans. Other important species of the Buthidae family are: *Androctonus crassicauda*, *Compsobuthus matthiesseni*, *Orthochirus* spp, *Mesobuthus eupeus*, *Odontobuthus doriae*, *Hottentotta schach*, *Ho. saulcyi*, *Mesobuthus caucasicus* and *Apistobuthus pterygocercus* (Malhotra et al. 1978, Radmanesh and Shaffiee 1989, Radmanesh 1990a,b, Mashak et al. 2000, Pipelzade 2007, Dehghani and Khamechian 2008, Dehghani et al. 2009, Jalali 2010, Dehghani and Fathi 2012, Dehghani et al. 2012).

Due to variability of scorpion venoms, the severity of envenoming is species dependent. Therefore, determination of the species responsible for sting is critical and can affect the clinical procedures of patient’s treatment. We recommend that the treatment for scorpion stinging in Iran should be based on the neurotoxic and cytotoxic effect of their venom produced in victims. It is necessary to distinguish all the native scorpion species, especially those medically important, in every region and determine their life, behaviors, mode of actions, and their venom properties (Dehghani et al. 2009, Dehghani and Fathi 2012). It is recommended to consider the possible connection between any stung patient and dangerous species of the region, for this it will be useful to investigate about the species of scorpion from the victims’ relative or those who accompany the patient. In addition, it is necessary that physicians and health care professionals are familiar with native scorpions, especially those that are potentially more dangerous than others, the effective treatment protocols, and supportive care.

## Conclusion

Scorpion stings have a seasonal pattern and mostly take place during the warmer months of the year. Therefore, formation of professional reinforcement staff and regular visits to high risk regions during this time can significantly reduce the risk of scorpion stings. Moreover, due to the rate of stings is higher in rural regions, training the rural people in public places like schools, mosques, and also by influenced persons will reduces the number of stings incidents.
